# Maximum Profit Output Configuration of Multi-Reservoir Resource Exchange Intermediary

**DOI:** 10.3390/e24101451

**Published:** 2022-10-11

**Authors:** Lingen Chen, Shaojun Xia

**Affiliations:** 1 Institute of Thermal Science and Power Engineering, Wuhan Institute of Technology, Wuhan 430205, China; 2 School of Mechanical & Electrical Engineering, Wuhan Institute of Technology, Wuhan 430205, China; 3 School of Power Engineering, Naval University of Engineering, Wuhan 430033, China

**Keywords:** multi-reservoir commercial engine, maximum profit, optimal control, finite time thermodynamics, generalized thermodynamic optimization

## Abstract

A model of a multi-reservoir resource exchange intermediary also defined as a commercial engine is proposed according to analogies and similarities between thermodynamics and economics. The optimal configuration of a multi-reservoir commercial engine with a maximum profit output objective is determined by applying optimal control theory. The optimal configuration consists of two instantaneous constant commodity flux processes and two constant price processes, and the configuration is independent of a number of economic subsystems and commodity transfer law qualitatively. The maximum profit output needs some economic subsystems to never contact with the commercial engine during commodity transfer processes. Numerical examples are provided for a three-economic-subsystem commercial engine with linear commodity transfer law. The effects of price changes of an intermediate economic subsystem on the optimal configuration of a three-economic-subsystem and the performance of optimal configuration are discussed. The research object is general, and the results can provide some theoretical guidelines for operations of actual economic processes and systems.

## 1. Introduction

Since the 1970s, Finite Time Thermodynamics (FTT) has made great progress in physics and engineering fields [1,2,3,4,5,6,7,8,9,10]. Many scholars have researched the optimal performances of thermal and chemical processes and cycles [11,12,13,14,15,16,17,18,19,20,21,22,23,24,25,26,27,28,29,30,31,32,33,34,35]. A great deal of work has been performed for the optimal configurations of thermal and chemical processes and cycles [36,37,38,39,40,41,42,43,44,45,46,47,48,49,50,51,52,53], including heat transfer, mass transfer, heat and mass transfer, heat engine, refrigerator, heat pump, chemical reaction, chemical engine, chemical pump, etc., with various optimization objectives and considering various transport laws. Amelkin et al. [44,45] proposed a multi-reservoir heat engine model and found the maximum power configuration. Xia et al. [46] proposed a multi-reservoir chemical engine model and found the maximum power configuration.

The analogies and similarities between thermodynamics and economics have been studied in some years. Some work was based on classical thermodynamics [47,48,49,50]. Tsirlin [51] applied the FTT idea and method into economics firstly. The finite commodity flow rate (CFR) was considered to obtain a minimal expense and maximum profit output (MPO) of resource exchange with linear commodity transfer law (CTL) [n∝Δ(P)]. De Vos [52,53] provided the concept of endoreversible economics and introduced a generalized CTL [$n\propto \Delta (P^{m})$]. Tsirlin [54], Tsirlin et al. [55,56,57], and Amelkin et al. [58] applied FTT into microeconomics and performed capital dissipation minimization (CDM), which is analogous to entropy generation minimization for thermal, mass and chemical systems. Amelkin [59] and Tsirlin [60] performed CDM with linear CTL and MPO of complex economic systems. Chen [61] and Xia et al. [62,63] performed MPO [61] and CDM [62,63] of a single resource exchange process with a generalized CTL. Recently, Tsirlin et al. [64] provided a comprehensive discussion on FTT in economics.

Temperature difference leads to heat transfer, and price difference leads to commodity flow, which is the similarity between the heat transfer and commodity trade processes. However, heat flows from high temperature to low temperature, while commodities flow from low price to high price, and each commodity flow is accompanied by a counter capital flow, which is the main difference between them. The similarities and differences between the optimizations of these two processes need to be further indicated. Based on the models of a multi-reservoir heat engine proposed in Refs. [44,45] and a multi-reservoir chemical engine proposed in Ref. [46], this paper will build a model of multi-reservoir resource exchange intermediary also named a commercial engine by methods of analogy and transplantation firstly, and it will further derive its optimal cycle configuration for MPO by applying FTT. This research work will further extend the FTT idea and method to applications of non-conventional thermodynamic fields, and it will enrich generalized thermodynamic optimization theory [3,65,66,67,68].

## 2. Modelling

**Figure 1:**
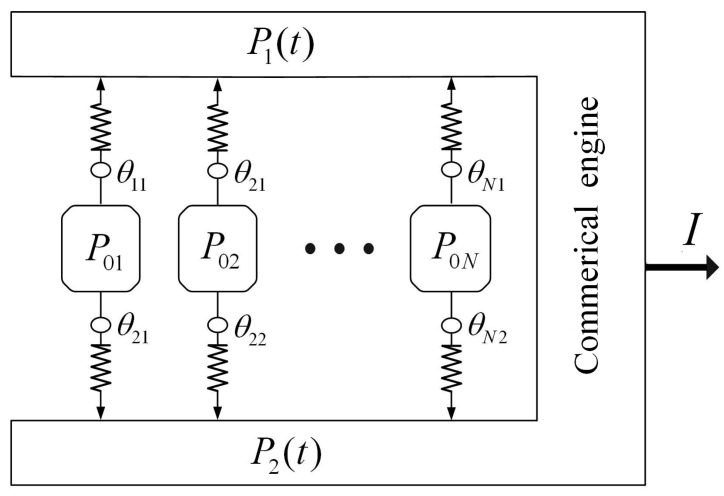
Model of a multi-reservoir commercial engine.

Figure 1 depicts a multi-reservoir commercial engine model. It consists of a commercial engine and *N* infinite economic subsystems. In the analysis and optimization of a thermodynamic system, the selection and boundary division of the research object, that is, the thermodynamic system, are very important. Similarly, in the analysis and optimization of economic systems, the selection and boundary division are also very important. The research object selected in this paper is the commercial engine: that is, the middleman or enterprise acting as the intermediary of commodity trading, and the commodity trading process between suppliers and consumers must be completed through the commercial engine rather than the direct trading process between suppliers and consumers. For example, there are situations where the commodity trading between suppliers and consumers cannot be completed directly due to the objective isolated geographical conditions or the imperfect trust mechanism. For the direct commodity exchange between suppliers and consumers, that is named products can be obtained more cheaply by parallel importing them from a different market from one’s own, this phenomenon also exists, which is not the research content of this paper. The specific research on the direct commodity exchange between suppliers and consumers can be seen in Refs. [61,62].

The purchased and sold prices of the commodity by the commercial engine are $P_{1}(t)$ and $P_{2}(t)$, respectively. The estimated prices of the commodity by *N* infinite economic subsystems are $P_{0i}$, where i∈[1,N]. The commodity flow between the commercial engine and economic subsystems has the form

(1) $$\begin{array}{cc} {\tilde{g}}_{ia}(P_{0i},P_{a},\theta_{ia})=\theta_{ia}g_{ia}(P_{0i},P_{a}), & i\in [1,N] \end{array}$$

where a∈{1,2}, ${\tilde{g}}_{ia}(P_{0i},P_{a},\theta_{ia})$ is practical CFR, and $g_{ia}(P_{0i},P_{a})$ is an ideal CFR. The contact function $\theta_{ia}$ describes the contact state between the economic subsystem and the commercial engine. If the commercial engine is fully contacted with the i-th economic subsystem, $\theta_{ia}$ = 1; if the commercial engine has no contact, $\theta_{ia}$ = 0. That is, $0\leq \theta_{ia}\leq 1$. All processes in economic subsystems and in commercial engine are reversible, and there is sole irreversibility in finite-rate commodity flow between economic subsystems and commercial engines. The ideal CFR $g_{ia}(P_{0i},P_{a})$ between a commercial engine and economic subsystem shows the standard behavior of CTL as a function of commodity prices $P_{0i}$ and $P_{a}$. Let the directions of the commodity flowing into and out of the commercial engine be positive and negative, respectively. As the commodity flows from low to high price, $g_{ia}(P_{0i},P_{a})<0$ if $P_{0i}>P_{a}$, $g_{ia}(P_{0i},P_{a})>0$ if $P_{0i}<P_{a}$, and $g_{ia}(P_{0i},P_{a})=0$ if $P_{0i}=P_{a}$. This is the main difference between the economic system in this paper and the thermodynamic system in Refs. [44,45]. The system operates under cyclic condition with a fixed duration, and there is no commodity cumulated inside the commercial engine

(2) $$\frac{1}{\tau }\int_{0}^{\tau }\sum_{i=1}^{N}[{\tilde{g}}_{i1}(P_{0i},P_{1},\theta_{i1})+{\tilde{g}}_{i2}(P_{0i},P_{2},\theta_{i2})]dt=0$$

The instantaneous profit Π of the commercial engine is

(3) $$\Pi (P_{0},P_{1}(t),P_{2}(t),\theta_{1}(t),\theta_{2}(t))=-\sum_{i=1}^{N}\left[{\tilde{g}}_{i1}(P_{0i},P_{1},\theta_{i1})P_{1}+{\tilde{g}}_{i2}(P_{0i},P_{2},\theta_{i2})P_{2}\right]$$

where $\theta_{a}$ is the contact function vector and $P_{0}$ is the economic subsystem price vector:

(4) $$\theta_{a}=(\theta_{1a},\theta_{2a},\ldots \ldots ,\theta_{Na})$$

(5) $$P_{0}=(P_{01},P_{02},\ldots \ldots ,P_{0N})$$

The average profit $\bar{\Pi }$ of the commercial engine per unit time over the total cycle is given by

(6) $$\bar{\Pi }=\frac{I}{\tau }=-\frac{1}{\tau }\int_{0}^{\tau }\sum_{i=1}^{N}\left[{\tilde{g}}_{i1}(P_{0i},P_{1},\theta_{i1})P_{1}+{\tilde{g}}_{i2}(P_{0i},P_{2},\theta_{i2})P_{2}\right]dt$$

where I is the total profit of the commercial engine over the total cycle.

## 3. Optimizing Configuration

The problem that should be solved now is to maximize the average profit of a multi-reservoir commercial engine within τ, that is, to determine the optimal time path of vector elements ($\theta_{a}(t)=(\theta_{1a},\theta_{2a},\ldots \ldots ,\theta_{Na})$) of contact functions as well as prices $P_{1}(t)$ and $P_{2}(t)$ of a commercial engine for the maximum $\bar{\Pi }$ shown by Equation (6) subject to the constraint shown by Equation (2). There are 2N+2 control variables, and the prices $P_{1}(t)$ and $P_{2}(t)$ satisfy the condition $0<P_{1}(t),P_{2}(t)<\infty$. The contact function vector $\theta_{a}(t)$ satisfies

(7) $$\begin{array}{ccc} 0\leq \theta_{ia}(t)\leq 1; & i\in [1,N], & a\in \{1,2\} \end{array}.$$

From the objective function of Equation (6) and constraint of Equation (2), the optimization problem is a typical averaged nonlinear programming problem. Therefore, a modified Lagrangian (*L*) is obtained

(8) $$L=-\sum_{i=1}^{N}\left[{\tilde{g}}_{i1}(P_{0i},P_{1},\theta_{i1})(P_{1}+\lambda )+{\tilde{g}}_{i2}(P_{0i},P_{2},\theta_{i2})(P_{2}+\lambda )\right]$$

where λ is the time-dependent Lagrange multiplier.

### 3.1. Optimal Contact Function Paths

From Equation (8), the Lagrangian *L* depends linearly on each control variable $\theta_{ia}$; therefore, the optimal values of $\theta_{ia}$ are the well-known “bang bang” solutions: that is, L will attain its maximum value only at boundary values {0,  1} of admissible $\theta_{ia}$. The Pontryagin maximum principle gives a rule of contact function:

(9) $$\theta_{ia}(P_{0i},P_{ia})=\left\{\begin{array}{cc} 0, & g_{ia}(P_{0i},P_{a})(P_{a}+\lambda )>0,\\ 1, & g_{ia}(P_{0i},P_{a})(P_{a}+\lambda )<0, \end{array}\right.\;\;i\in [1,N],\;a\in \{1,2\}$$

Taking a closer look at Equation (9), for $g_{ia}(P_{0i},P_{a})<0$, it implies that contact $P_{a}$ of a commercial engine connects with the economic subsystem at high price and sells the commodity to it, thus fulfilling the $P_{0i}>P_{a}$ condition. While for $g_{ia}(P_{0i},P_{a})>0$, it implies the $P_{0i}<P_{a}$ condition. The commercial engine then contacts to the low-price economic subsystem and purchases the commodity from it. According to the span of the Lagrangian multiplier λ, three possible cases are distinguished as follows:

(1) When $\lambda >-P_{l}>-P_{h}$, one has

(10) $$(P_{h}+\lambda )>0\Rightarrow \left\{\begin{array}{l} \theta_{ih}(P_{0i},P_{h},\lambda )=1,\;\;\mathrm{if}\;g_{ih}<0,\;\mathrm{i.e.,}\;P_{0i}>P_{h};\;\\ \theta_{ih}(P_{0i},P_{h},\lambda )=0,\;\;\mathrm{if}\;g_{ih}\geq 0,\;\mathrm{i.e.,}\;P_{0i}\leq P_{h}. \end{array}\right.$$

(11) $$(P_{l}+\lambda )>0\Rightarrow \left\{\begin{array}{l} \theta_{il}(P_{0i},P_{l},\lambda )=1,\;\;\mathrm{if}\;g_{il}<0,\;\mathrm{i.e.,}\;P_{0i}>P_{l};\;\\ \theta_{il}(P_{0i},P_{l},\lambda )=0,\;\;\mathrm{if}\;g_{il}\geq 0,\;\mathrm{i.e.,}\;P_{0i}\leq P_{l}. \end{array}\right.$$

From Equations (10) and (11), all ${\tilde{g}}_{ia}$ are either negative or vanishing in this case due to that ${\tilde{g}}_{ia}(P_{0i},P_{a},\theta_{ia})=\theta_{ia}g_{ia}(P_{0i},P_{a})$. Further from the conservation law of amount of Equation (2), all ${\tilde{g}}_{ia}$ values have to be zero, and no profit is produced. This case is excluded from further consideration.

(2) When $-P_{l}>-P_{h}>\lambda$, one has

(12) $$(P_{h}+\lambda )<0\Rightarrow \left\{\begin{array}{l} \theta_{ih}(P_{0i},P_{h},\lambda )=0,\;\;\mathrm{if}\;g_{ih}<0,\;\mathrm{i.e.,}\;P_{0i}>P_{h};\;\\ \theta_{ih}(P_{0i},P_{h},\lambda )=1,\;\;\mathrm{if}\;g_{ih}\geq 0,\;\mathrm{i.e.,}\;P_{0i}\leq P_{h}. \end{array}\right.$$

(13) $$(P_{l}+\lambda )<0\Rightarrow \left\{\begin{array}{l} \theta_{il}(P_{0i},P_{l},\lambda )=0,\;\;\mathrm{if}\;g_{il}<0,\;\mathrm{i.e.,}\;P_{0i}>P_{l};\;\\ \theta_{il}(P_{0i},P_{l},\lambda )=1,\;\;\;\mathrm{if}\;g_{il}\geq 0,\;\mathrm{i.e.,}\;P_{0i}\leq P_{l}. \end{array}\right.$$

From Equations (12) and (13), all ${\tilde{g}}_{ia}$ are either positive or vanishing in this case due to that ${\tilde{g}}_{ia}(P_{0i},P_{a},\theta_{ia})=\theta_{ia}g_{ia}(P_{0i},P_{a})$. Further from the conservation law of amount of Equation (2), all ${\tilde{g}}_{ia}$ values have to be zero, and no profit is produced. This case is also excluded from further consideration.

(3) When $-P_{l}>\lambda >-P_{h}$, one has

(14) $$(P_{h}+\lambda )>0\Rightarrow \left\{\begin{array}{l} \theta_{ih}(P_{0i},P_{h},\lambda )=1,\;\;\mathrm{if}\;g_{ih}<0,\;\mathrm{i.e.,}\;P_{0i}>P_{h};\;\\ \theta_{ih}(P_{0i},P_{h},\lambda )=0,\;\;\mathrm{if}\;g_{ih}\geq 0,\;\mathrm{i.e.,}\;P_{0i}\leq P_{h}. \end{array}\right.$$

(15) $$(P_{l}+\lambda )<0\Rightarrow \left\{\begin{array}{l} \theta_{il}(P_{0i},P_{l},\lambda )=0,\;\;\mathrm{if}\;g_{il}<0,\;\mathrm{i.e.,}\;P_{0i}>P_{l};\;\\ \theta_{il}(P_{0i},P_{l},\lambda )=1,\;\;\mathrm{if}\;g_{il}\geq 0,\;\mathrm{i.e.,}\;P_{0i}\leq P_{l}. \end{array}\right.$$

From Equation (14), all ${\tilde{g}}_{ih}=g_{ih}(P_{0i},P_{h})$ are negative in this case due to that ${\tilde{g}}_{ia}(P_{0i},P_{a},\theta_{ia})=\theta_{ia}g_{ia}(P_{0i},P_{a})$, and this shows that the commercial engine sells commodity to economic subsystems with price $P_{0i}>P_{h}$. From the conservation law of amount (i.e., Equation (2)) and Equation (15), one can conclude that ${\tilde{g}}_{il}=g_{il}(P_{0i},P_{l})$ are all positive, and this shows that the commercial engine purchases commodity from economic subsystems with price $P_{0i}<P_{l}$.

Optimal contact functions show that an economic subsystem can be connected to one side of the commercial engine at most. The economic subsystems connected with the low-price side of the commercial engine sell the commodity to the commercial engine, and those connected with the high-price side of the commercial engine purchase the commodity from the commercial engine, while those with prices in the range between $P_{l}$ and $P_{h}$ are never connected with the commercial engine during a cycle. The set of N economic subsystems can be divided into three subsets: low-price, high-price, and unused economic subsystem sets, respectively. The unused economic subsystem set can be empty depending on its commodity price. The highest and lowest price economic subsystems are always active in a finite profit production solution.

### 3.2. Optimal Prices $P_{h}$ and $P_{l}$ for the Commercial Engine

The commodity transfer function for each economic subsystem is separated into the commodity input function and output function, that is, $g_{il}{}^{+}(P_{0i},P_{l})$ and $g_{ih}{}^{-}(P_{0i},P_{h})$, which are, respectively, given by

(16) $$g_{il}{}^{+}(P_{0i},P_{l})=\left\{\begin{array}{l} g(P_{0i},P_{l}),\;\;\;\;\;\mathrm{if}P_{0i}<P_{l}\\ 0,\;\;\;\;\;\;\;\;\;\;\;\;\;\;\;\;\;\;\;\mathrm{if}\;\;P_{0i}\geq P_{l} \end{array}\right.\;\;\;\;\;\;\;\;\;\;\;\;\;\;i\in [1,N]$$

(17) $$g_{ih}{}^{-}(P_{0i},P_{h})=\left\{\begin{array}{l} 0\;\;\;\;\;\;\;\;\;\;\;\;\;\;\;\;\;\;\;\;\;\mathrm{if}P_{0i}\geq P_{h}\\ g(P_{0i},P_{h}),\;\;\;\;\mathrm{if}\;P_{0i}<P_{h} \end{array}\right.\;\;\;\;\;\;\;\;\;\;\;\;\;\;\;\;\;\;\;\;\;\;i\in [1,N]$$

The total commodity rate input to and output from the commercial engine are the sum of all contributions $g_{il}{}^{+}(P_{0i},P_{l})$ and $g_{ih}{}^{-}(P_{0i},P_{h})$, that is, $g^{+}(P_{0},P_{l})$ and $g^{-}(P_{0},P_{h})$, which are, respectively, given by

(18) $$g^{+}(P_{0},P_{l})=\sum_{i=1}^{N}g_{il}{}^{+}(P_{0i},P_{l}),\;\;\;g^{-}(P_{0},P_{h})=\sum_{i=1}^{N}g_{ih}{}^{-}(P_{0i},P_{h})$$

The flow of the commodity occurs with those of money at the same time, and the total money flow rates used to purchase and obtained from selling the commodity by the commercial engine are denoted as $M^{-}(P_{0},P_{l})$ and $M^{+}(P_{0},P_{l})$, respectively, that is:

(19) $$M^{-}(P_{0},P_{l})=g^{+}(P_{0},P_{l})*P_{l},\;M^{+}(P_{0},P_{h})=g^{-}(P_{0},P_{h})*P_{h}$$

Substituting Equation (18) into Equation (8) yields

(20) $$L=-[g^{+}(P_{0},P_{l})(P_{l}+\lambda )+g^{-}(P_{0},P_{h})(P_{h}+\lambda )]$$

From $\partial L/\partial P_{l}=0$ and $\partial L/\partial P_{h}=0$, one has

(21) $$\lambda =-[\frac{\partial g^{+}(P_{0},P_{l})}{\partial P_{l}}P_{l}+g^{+}(P_{0},P_{l})]/[\frac{\partial g^{+}(P_{0},P_{l})}{\partial P_{l}}]$$

(22) $$\lambda =-[\frac{\partial g^{-}(P_{0},P_{h})}{\partial P_{h}}P_{h}+g^{-}(P_{0},P_{h})]/[\frac{\partial g^{-}(P_{0},P_{h})}{\partial P_{h}}]$$

For the give commodity transfer law $g_{i}(P_{0i},P)$ and estimate commodity price $P_{0i}$ of the economic subsystem, the values of parameters $P_{h}$, $P_{l}$ and λ are obtained by combining Equation (2) together with Equations (21) and (22).

## 4. Numerical Examples and Discussions

A commercial engine with three economic subsystems is considered as an example herein. The estimate commodity prices of the three economic subsystems are $P_{01}$, $P_{02}$, and $P_{03}$, respectively. They could either purchase the commodity from the commercial engine or sell the commodity to the commercial engine. The CFR is assumed to obey the linear CTL:

(23) $$g_{i}(P_{0i},P)=\alpha_{i}(P_{0i}-P)$$

**Figure 2:**
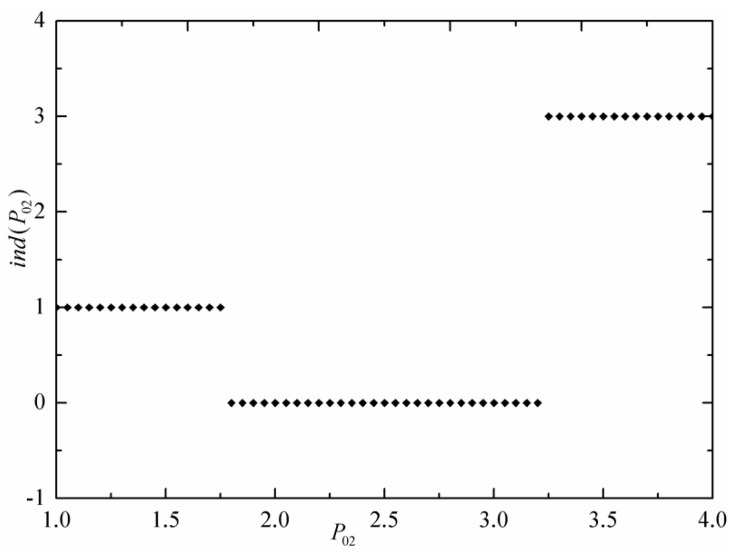
Indicator function vs. estimate commodity price of intermediate economic subsystem.

**Figure 3:**
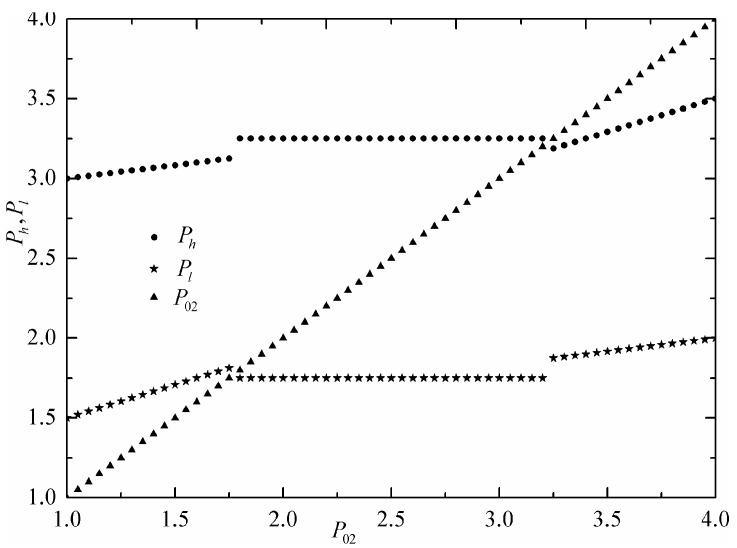
Commodity prices of commercial engine vs. estimate commodity price of intermediate economic subsystem.

The lowest and highest commodity prices of three economic subsystems are fixed as $P_{03}=4$ and $P_{01}=1$. Without loss of generality, $\alpha_{1}=\alpha_{2}=\alpha_{3}=1$ are set by selecting appropriate units. Figure 2 shows the indicator function $ind(P_{02})$ versus commodity price of the intermediate economic subsystem. Figure 3 shows the commodity prices ($P_{h}$) and $P_{l}$ of the commercial engine versus commodity price ($P_{02}$) of the intermediate economic subsystem. For low price $P_{02}$ that satisfies $P_{02}<P_{l}$ in Figure 3, both the economic subsystems 1 and 2 sell the commodity to the commercial engine as the low-price economic subsystem; therefore, the indicator function $ind(P_{02})$ is 1 in Figure 2. For high price $P_{02}$ that satisfies $P_{02}>P_{h}$ in Figure 3, the intermediate economic subsystem 2 is in contact with commercial at the same time as economic subsystem 3; therefore, the indicator function $ind(P_{02})$ is 3 in Figure 2. For high price $P_{02}$ that satisfies $P_{l}<P_{02}<P_{h}$ in Figure 3, intermediate economic subsystem 2 is not used; therefore, indicator function $ind(P_{02})$ is 0 in Figure 2. The economic reason is that for the multi-reservoir commercial engine considered herein, this unused intermediate economic subsystem is neither low-price commodity supplier nor high-price commodity consumer, and therefore, it is useless for the commercial engine to produce profit.

**Figure 4:**
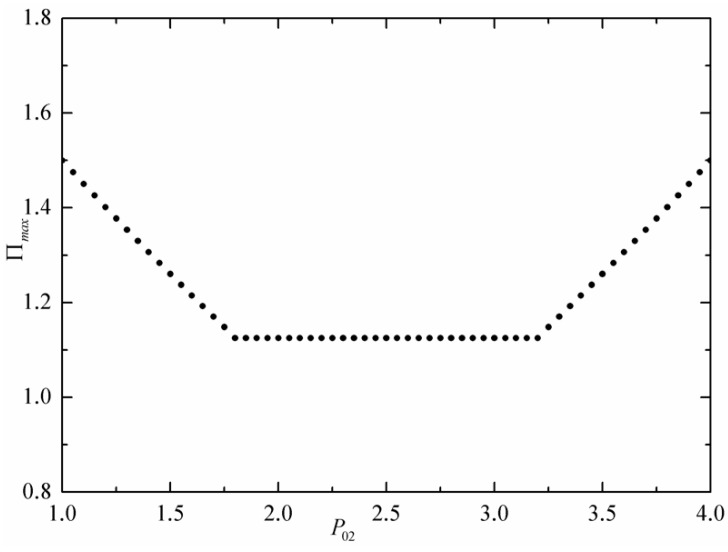
MPO per unit time vs. estimate commodity price intermediate economic subsystem.

Figure 4 shows the resulting MPO ($\Pi_{\max}$) per unit time versus the commodity price of the intermediate economic subsystem 2. When $P_{02}$ increases, MPO decreases until the intermediate economic subsystem 2 is switched off; then, it remains constant, and finally, the intermediate economic subsystem 2 is switched on again, and MPO increases again. For the price $P_{02}$ in the range between $P_{02}=P_{l}$ and $P_{02}=P_{h}$ in Figure 4, MPO ($\Pi_{\max}$) per unit time of the commercial engine achieves its minimum value.

**Figure 5:**
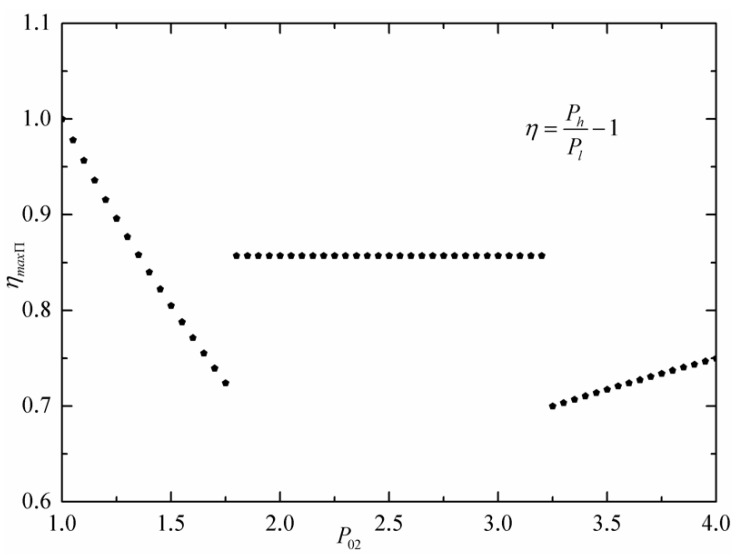
Efficiency at MPO vs. estimate commodity price of intermediate economic subsystem.

The general definition of economic index is the ratio of gain to cost. In thermodynamics, the economic index of a heat engine is the thermal efficiency, which is defined as the ratio of the network output of the cycle to the heat absorbed from the high-temperature heat source. The economic index of a reversible Carnot engine cycle is Carnot efficiency, which is the upper limit of thermal power conversion efficiency between the high-temperature heat source and low-temperature heat sink with the same temperature limits. Similarly, in economics, the economic index of a commercial machine is economic efficiency, that is, profit rate, which is defined as the ratio of the net profit obtained by the commercial engine to the cost paid for purchasing commodity from the supplier. Similar to the reversible Carnot engine, the economic efficiency of the reversible Carnot commercial engine is the upper limit of the economic efficiency of a commercial engine between low-price supplier and high-price consumer with the same price limits. The economic efficiency of the commercial engine is defined as $\eta =(P_{h}-P_{l})/P_{l}=P_{h}/P_{i}-1$. Figure 5 shows efficiency ($\eta_{\max\Pi }$) at MPO versus commodity price $P_{02}$ of an intermediate economic subsystem. When $P_{02}$ increase and satisfies $P_{02}<P_{l}$, $\eta_{\max\Pi }$ decreases; when $P_{02}$ increases and satisfies $P_{l}<P_{02}<P_{h}$, $\eta_{\max\Pi }$ is constant. When $P_{02}$ increases and satisfies $P_{h}<P_{02}$, that is, intermediate economic subsystem 2 is switched on again, $\eta_{\max\Pi }$ increases.

## 5. Conclusions

Based on the models of a multi-reservoir heat engine proposed in Refs. [44,45] and a multi-reservoir chemical engine proposed in Ref. [46], this paper proposes a model of a multi-reservoir commercial engine by methods of analogy and transplantation firstly, and it further derives its optimal cycle configuration for MPO by applying FTT. Numerical examples are provided for a three-economic-subsystem commercial engine with linear CTL; the MPO and its corresponding efficiency are provided. The results show that:

1. Optimal configuration consists of two instantaneous constant commodity flux processes and two constant price processes, where the used economic subsystems and the profit-producing commercial engine contact prices are time-independent, and the configuration is independent of number of economic subsystems and CTL qualitatively. Different CTLs have no influence on the optimal configuration of commercial engine qualitatively, but only quantitatively. Effects of different CTLs on the multi-reservoir commercial engine performance will be our next research work.
2. For attaining MPO, some economic subsystems should never come into contact with the commercial engine during commodity transfer processes. These unused subsystems are referred to as unused subsystems. The highest price consumer and the lowest price supplier will always be used. This shows that in order to obtain a favorable market survival environment under competitive conditions, commodity suppliers should take positive and effective measures to reduce the manufacturing cost of commodities and then reduce the selling price of commodities, so as to become the lowest price economic subsystem. In addition, commodity consumers should take active and effective measures to improve the utility and value of commodities so as to improve the purchase price of commodities and become the highest price economic subsystem.
3. A multi-reservoir commercial engine is more general than a common two-reservoir commercial engine, and the results can provide theoretical guidelines for the optimal operation of actual economic processes.

## Data Availability

Not applicable.

## Nomenclature

*g*	ideal commodity flow rate
$\tilde{g}$	practical commodity flow rate
*I*	total profit
*L*	modified Lagrangian function
m	power index related to commodity transfer law
*N*	the economic subsystem number
n	commodity flow rate
*P*	price
*t*	time
**Greek symbols**	
α	commodity flow coefficient
η	economic efficiency
λ	Lagrange multiplier
θ	contact function
τ	cycle period
$\bar{\Pi }$	average profit
**Subscripts**	
0*i*	the *i*-th economic subsystem
1	purchased price
2	sold price
h	high price
i	number
l	low price
max	maximum
0i	the i-th economic subsystem
1	purchased price
**Superscripts**	
+	input
−	output
**Abbreviations**	
CDM	capital dissipation minimization
CFR	commodity flow rate
CTL	commodity transfer law
FTT	finite time thermodynamics
ind	index
MPO	maximum profit output
